# Expression of a Functional Mx1 Protein Is Essential for the Ability of RIG-I Agonist Prophylaxis to Provide Potent and Long-Lasting Protection in a Mouse Model of Influenza A Virus Infection

**DOI:** 10.3390/v14071547

**Published:** 2022-07-15

**Authors:** Lara S. U. Schwab, Fernando Villalón-Letelier, Melkamu B. Tessema, Sarah L. Londrigan, Andrew G. Brooks, Aeron Hurt, Christoph Coch, Thomas Zillinger, Gunther Hartmann, Patrick C. Reading

**Affiliations:** 1 Department of Microbiology and Immunology, The University of Melbourne at the Peter Doherty Institute for Infection and Immunity, 792 Elizabeth St, Melbourne, VIC 3000, Australia; lara.schwab@unimelb.edu.au (L.S.U.S.); fernandojavier.villalon@mssm.edu (F.V.-L.); melkamubezie.tessema@unimelb.edu.au (M.B.T.); sarahll@unimelb.edu.au (S.L.L.); agbrooks@unimelb.edu.au (A.G.B.); 2 Institute of Clinical Chemistry and Clinical Pharmacology, University Hospital Bonn, 53127 Bonn, Germany; ccoch@gmx.net (C.C.); zillinger@uni-bonn.de (T.Z.); gunther.hartmann@uni-bonn.de (G.H.); 3 WHO Collaborating Centre for Reference and Research on Influenza, Victorian Infectious Diseases Reference Laboratory at the Peter Doherty Institute for Infection and Immunity, 792 Elizabeth St, Melbourne, VIC 3000, Australia; aeron.hurt@roche.com

**Keywords:** influenza, RIG-I, Mx, innate immunity, mouse model

## Abstract

RIG-I is an innate sensor of RNA virus infection and its activation induces interferon-stimulated genes (ISGs). In vitro studies using human cells have demonstrated the ability of synthetic RIG-I agonists (3pRNA) to inhibit IAV replication. However, in mouse models of IAV the effectiveness of 3pRNA reported to date differs markedly between studies. Myxoma resistance (Mx)1 is an ISG protein which mediates potent anti-IAV activity, however most inbred mouse strains do not express a functional Mx1. Herein, we utilised C57BL/6 mice that do (B6.A2G-*Mx1*) and do not (B6-WT) express functional Mx1 to assess the ability of prophylactic 3pRNA treatment to induce ISGs and to protect against subsequent IAV infection. In vitro, 3pRNA treatment of primary lung cells from B6-WT and B6.A2G-*Mx1* mice resulted in ISG induction however inhibition of IAV infection was more potent in cells from B6.A2G-*Mx1* mice. In vivo, a single intravenous injection of 3pRNA resulted in ISG induction in lungs of both B6-WT and B6.A2G-*Mx1* mice, however potent and long-lasting protection against subsequent IAV challenge was only observed in B6.A2G-*Mx1* mice. Thus, despite broad ISG induction, expression of a functional Mx1 is critical for potent and long-lasting RIG-I agonist-mediated protection in the mouse model of IAV infection.

## 1. Introduction

Influenza A virus (IAV) infections are associated with substantial mortality and morbidity, particularly amongst vulnerable groups such as the elderly and the immunocompromised. New approaches are urgently required to provide protection against IAV infection, as well as to treat established infections. Over the last decade, substantial progress has been made towards developing new therapeutic options, including drugs that activate particular components of innate immunity which have the potential to elicit broad antiviral responses [1]. The innate immune system represents a first line of host defence against invading pathogens, including viruses, with pattern recognition receptors (PRRs) recognizing “pathogen-associated molecular patterns” (PAMPs) to elicit cell-intrinsic immunity [2]. For example, retinoic acid-inducible gene I (RIG-I) is expressed by most nucleated cells and recognises blunt-end double-stranded RNA (dsRNA) with a 5′-triphosphate or 5′-diphosphate [3,4,5]. Following RIG-I activation, intracellular signaling pathways ultimately result in the production of type I and type III interferons (IFNs) as well as pro-inflammatory cytokines. Following secretion, type I and III IFNs bind to their cognate receptors in an autocrine and/or paracrine manner to elicit intracellular signaling pathways and induce expression of hundreds of IFN-stimulated genes (ISGs) [6]. This, in turn, results in the induction of an ‘antiviral state’ within infected cells, as well as in surrounding uninfected cells, thereby limiting virus replication and spread.

Growing evidence indicates that synthetic RIG-I agonists can act as potent antiviral agents against diverse viruses in vitro, including IAV, human immunodeficiency virus (HIV)-1, hepatitis C virus (HCV), dengue virus (DENV), chikungunya virus (CHIKV), vesicular stomatitis virus (VSV), and vaccinia virus [7,8,9,10]. RIG-I agonists are 5′-triphosphate RNA oligomers and are generated as previously described [11]. Most in vitro studies have assessed the antiviral activity of RIG-I agonists using human cells and/or cell lines. For example, pre-treatment of human A549 cells [8,9,10] or monocyte-derived dendritic cells [9] with RIG-I agonists resulted in reduced intracellular expression of IAV proteins and/or reduced titres of infectious virus following subsequent IAV infection. Several studies have also used mouse models to demonstrate the prophylactic and/or therapeutic effects of RIG-I agonists in vivo against a smaller subset of viruses, namely IAV [8,9,10,11], CHIKV [7] and SARS-CoV-2 [12,13].

In mouse models of IAV, most studies have examined the impact of intravenous (i.v.) pre-treatment of inbred C57BL/6 or Balb/c mouse strains with one or more doses of RIG-I agonist prior to IAV infection [8,9,10], often with subsequent i.v. doses at the time of and/or at various times after experimental infection. Of note, up to 4 i.v. doses of RIG-I agonist are well tolerated and do not induce adverse reactions in mice [11]. Moreover, it is clear that i.v. treatment of mice with RIG-I agonists can provide protection against IAV infection, resulting in improved survival and/or reduced titres of virus in the lungs [8,9,10].

Mx proteins are dynamin-like GTPases and well-characterized IFN-induced antiviral effector proteins (reviewed in [14]). The importance of mouse Mx1 and human MxA has been particularly well studied by using Mx1-congenic and MxA-transgenic mice which show marked resistance to IAV infection [15,16,17,18]. While functional Mx proteins are expressed in wild mice, most of the commonly used laboratory mouse strains, including C57BL/6 or Balb/c mice, have lost expression of a functional Mx1 protein and are therefore more susceptible to experimental IAV infection [19]. To date, most studies assessing the impact of synthetic RIG-I agonists on IAV infection in vivo have utilized C57BL/6 [8] or Balb/c [9,10] mice to demonstrate relatively modest protective effects following subsequent IAV challenge. However, Coch et al. demonstrated that even a single 3pRNA treatment of C57BL/6 mice expressing a functional Mx1 protein resulted in potent inhibition of IAV-induced weight loss, improved survival rate and improved clinical disease score [11]. More, prophylaxis of these mice with 3pRNA even 7 days prior to IAV challenge still resulted in potent protection from IAV-induced weight loss and disease.

Given the remarkable differences in potency of 3pRNA treatment protecting against IAV infection of mice observed in the study by Coch et al. [11] compared to previous reports [8,9,10], we aimed to directly compare the impact of 3pRNA prophylaxis on subsequent IAV infection using inbred congenic C57BL/6 mice that do (B6.A2G-*Mx1*) and do not (B6-WT) express a functional Mx1 protein. Homozygous B6.A2G-*Mx1* and B6-WT mouse lines were derived from breeding of heterozygous littermates and therefore differed genetically only in regard to expression of Mx. First, we demonstrated that pre-treatment with a synthetic RIG-I resulted in potent ISG induction in mouse airway cells, including primary lung fibroblasts from B6-WT and B6.A2G-*Mx1* mice. Moreover, ISG induction following RIG-I agonist treatment correlated with reduced IAV infection and growth following subsequent infection with IAV in vitro. In vivo, we demonstrated that a single prophylactic dose of 3pRNA via the i.v. route resulted in more effective protection against subsequent IAV infection than an equivalent dose via the intranasal (i.n.) route. Moreover, while i.v. 3pRNA results in modest protection of B6-WT mice when challenged 24 h later with IAV, only B6.A2G-*Mx1* mice showed potent and long-lasting protection when challenged with IAV 1 or 5 days after prophylaxis. Together, these data define the critical importance of RIG-I agonist-mediated induction of Mx1 for potent and long-lasting protection against IAV in the mouse model.

## 2. Materials and Methods

### 2.1. Cells and Viruses

Human lung epithelial (A549) cells or primary mouse lung fibroblasts (MLF, isolated as described [20]) were maintained and passaged in DMEM (Gibco) containing 10 % (vol/vol) fetal calf serum (FCS) (Sigma-Aldrich, St. Louis, MO, USA) and supplemented with 2 mM L-glutamine (Gibco), 1 mM sodium pyruvate (Gibco), 100 U/mL penicillin, and 100 μg/mL of streptomycin (Gibco). Mouse lung epithelial (LA-4) cells were cultured in Ham’s F-12K (Kaighn’s)-Medium (Gibco), supplemented as above. Madin-Darby canine kidney (MDCK) cells (ATCC CCL-34) were maintained and passaged in RPMI 1640 medium (Gibco) supplemented with 10 % (*v/v*) FCS, 2 mM L-glutamine, 100 Units/mL of penicillin, 100 μg/mL of streptomycin and 1 mM sodium pyruvate.

### 2.2. Influenza A Virus (IAV)

IAV strains used in this study were A/Perth/265/2009 (H1N1pdm09), A/Puerto Rico/8/1934 (PR8, H1N1), and A/HKx31 (HKx31, H3N2), a high-yielding reassortant of A/Aichi/2/68 (H3N2) with PR8 bearing the H3N2 surface glycoproteins. Viruses were obtained from the Department of Microbiology and Immunology, The University of Melbourne (DMI UoM), Melbourne, Australia or from the WHO Collaborating Centre for Reference and Research on Influenza (WHO CCRRI), Melbourne, Australia). Viruses were generally propagated in the allantoic cavity of 10-day embryonated chicken eggs following standard procedures [21] and titres of infectious virus were determined on MDCK cells by standard plaque assay and expressed as plaque-forming units (PFU) per mL [22]. However, stocks of A/Perth/265/2009 (H1N1pdm09) were propagated in MDCK cells following standard procedures [23] and virus titres were determined on MDCK cells by 50 % tissue culture infectious dose (TCID50) assay and expressed as TCID50/mL [24] or determined by standard plaque assay on MDCK cells as described [22].

### 2.3. Oligonucleotides and Transfection

3pRNA was prepared as previously described [11]. CA21-mer oligonucleotide (5′-CACACACACACACACACACAC-3′) was used as control RNA as previously described [13]. For in vitro studies, Lipofectamine 2000 (Invitrogen) was used to transfect mammalian cells with 3pRNA (100 ng/mL) or ctrl RNA (100 ng/mL) in OptiMEM (Gibco-BRL) according to manufacturer’s instructions.

### 2.4. In Vitro Virus Infection and Growth Assays

After overnight culture, IAV was added to cell monolayers in serum-free medium at the indicated multiplicity of infection (MOI) and incubated for 1 h at 37 °C. Cells were washed and incubated an additional 7 h in serum-free medium then fixed, permeabilised and stained for the viral nucleoprotein (NP) of IAV (D67J, Thermo Fisher Scientific, Waltham, MA, USA). Fixable viability dye eFluor 780 (eBioscience) was included to exclude dead cells. For virus growth assays, after overnight culture cells were infected with a low MOI of IAV in serum-free medium for 1 h in the presence of 0.5 μg/mL TPCK-treated trypsin (Worthington Biochemical, Lakewood, NJ, USA) to facilitate cleavage of viral HA0 and therefore multiple cycles of virus replication. Cells were washed, cultured for various times in serum-free media in the presence of trypsin and supernatants were harvested at indicated time points and virus titres were determined in clarified supernatants by VS assay. Titres were expressed as Virospot (VS)/mL.

### 2.5. Animal Models

All research complied with the University of Melbourne’s Animal Experimentation Ethics guidelines and policies in accordance with the NHMRC Australian code of practice for the care and use of animals for scientific purposes.

### 2.6. Plaque and Virospot Assays to Determine Titers of Infectious IAV

Titers of infectious IAV in culture supernatants were determined by Virospot assay on MDCK cells as described [25]. Titers of infectious IAV in clarified tissue homogenates from IAV-infected mice were determined by standard plaque assay on MDCK cells as described [22].

### 2.7. In Vivo Treatment and Infection of Mice

Congenic B6.A2G-*Mx1* mice carrying the intact A2G wild-type *Mx1* resistance allele on a C57BL/6 background have been described [26]. Heterozygote animals were bred to obtain homozygous animals expressing functional (B6.A2G-*Mx1*) or non-functional Mx1 (B6-WT) for use in experiments or for further breeding. Homozygous breeders were used for 2–4 litters and then replaced with new breeders obtained from heterozygous matings. Animals were bred and maintained in specific pathogen-free conditions at the Bioresources Facility at The Peter Doherty Institute for Infection and Immunity, Melbourne, Australia. 12.5 μg of 3pRNA or ctrl RNA was formulated in in vivo jetPEI transfection reagent (Polyplus-transfection, France) at a N/P ratio of 8 and intravenously injected. The N/P ratio is a measure of the ionic balance within the RNA complexes and is defined as the number of nitrogen residues of in vivo-jetPEI per nucleic acid phosphate. At the indicates timepoints, mice were infected with the indicated dose of IAV in 50 μL of PBS via the intranasal route.

### 2.8. Gene Expression Analysis by Quantitative Real Time PCR

Total RNA was isolated from PBMCs or cell lines using RNeasy Mini Kit (Qiagen, Hilden, Germany) according to manufacturer’s instructions, including on-column DNase treatment (Qiagen), and then reverse transcribed into cDNA using the Omniscript RT Kit (Qiagen). Real-time PCR reactions were performed using Sensifast Lo-ROX SYBR Green (Bioline, Toronto, ON, Canada) on the ABI7500 Real Time PCR System (Applied Biosystems, Waltham, MA, USA) using specific primers for murine ISG15 [27], murine GAPDH [28] and murine IFIT1 [29].

Primers for human IFITM1 (fwd: AGCATTCGCCTACTCCGTGAAG, rev: CACAGAGCCGAATACCAGTAACAG) and the housekeeping GAPDH (fwd: TGAAGGTCGGAGTCAACGG, rev: GGCAACAATATCCACTTTACCAGAG) were used. Gene expression was normalized to housekeeping gene GAPDH and graphed as fold change to untreated controls using 2^−ΔΔCT^ method [30].

### 2.9. Statistical Analysis

Statistical analyses were conducted using Prism version 8.0 and are described in the figure legends.

## 3. Results

### 3.1. RIG-I Agonist Treatment Induces ISG Expression and Inhibits IAV Infection and Growth in Human and Mouse Airway Cell Lines

**Figure 1:**
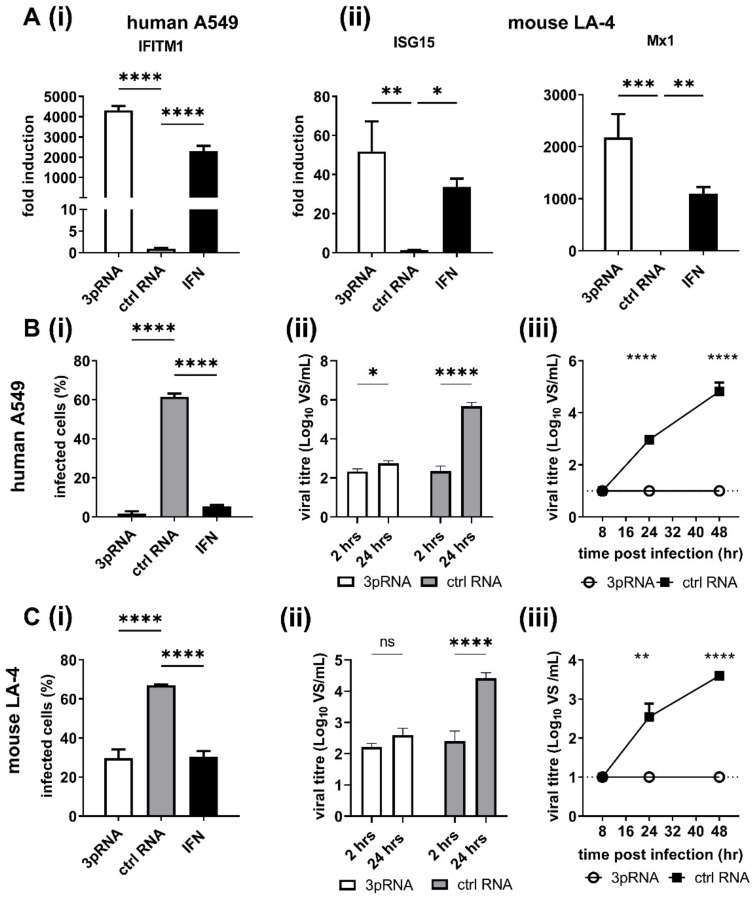
Treatment of human and mouse airway cell lines with RIG-I agonist induces ISG expression and inhibits IAV infection and replication. LA-4 and A549 cells were transfected with RIG-I agonist (3pRNA, 200 ng/mL) or control RNA (ctrl RNA, 200 ng/mL), or incubated with human (10^4^ units/mL) or mouse (10^4^ units/mL) IFN-α. (**A**) After 24 h, total RNA was isolated and expression of ISGs assessed by qRT-PCR. Expression is normalized to GAPDH and expressed as fold induction relative to untreated cells. (**B**) human A549 or (**C**) mouse LA-4 cells, were (i) inoculated for 1 h at 37 °C with IAV (strain HKx31) at MOI of 5 (A549) or 10 (LA-4). Cells were incubated an additional 7 h, then fixed and stained for intracellular expression of the viral NP and analysed by flow cytometry. (ii) Cells were inoculated for 1 h with HKx31 MOI of 5 (A549) or 10 (LA-4), washed and cell-free supernatant was harvested at 2 hpi or 24 hpi. (iii) Cells were incubated at 37 °C in the presence of exogenous trypsin (0.5 mg/mL). In (**B**,**C**) (ii) and (iii) titres of infectious virus were determined in clarified supernatants at various times post-infection using a VS assay on MDCK cells. All data show the mean (±SD) from triplicate samples and are representative of 2 or more independent experiments except for (**C**) (ii), where one experiment in triplicates was performed. A one-way ANOVA with Bonferroni’s multiple comparison test was used in (**A**),(**B**(i)) and (**C**(i)) and a Student’s unpaired *t*-test was used in (**B**(ii),(iii)) and (**C**(ii),(iii)) to compare 3pRNA or ctrl RNA treatment. * = *p* < 0.05; ** = *p* < 0.01; *** = *p* < 0.001; **** = *p* < 0.0001.

Previous studies using human A549 airway epithelial cells demonstrated that RIG-I agonists are potent inhibitors of IAV replication [8,9,10,31]. Given that mice represent an important preclinical model to investigate the effectiveness of antiviral treatments, including the ability of RIG-I agonist to inhibit IAV infections, we compared ISG induction in human A549 and mouse LA-4 airway epithelial cells following treatment with RIG-I agonist. Therefore, cells were treated with RIG-I agonist (3pRNA) or with a control RNA that does not activate RIG-I (ctrl RNA) and ISG induction was assessed 24 h later. Recombinant human or mouse IFN-α was also used to treat A549 or LA-4 cells, respectively. As seen in Figure 1A, 3pRNA or IFN-α treatment induced potent upregulation of ISGs in both human (IFITM1) and mouse (ISG15, Mx1) airways cell lines, whereas ctrl RNA treatment did not. Of note, LA-4 cells express the *Mx1* gene but do not express a functional Mx protein.

Next, A549 (Figure 1B) or LA-4 cells (Figure 1C) were pre-treated with ctrl RNA, 3pRNA or species-specific IFN-α and then infected with IAV strain HKx31 (H3N2) and the percentage of IAV-infected cells was determined at 8 h post-infection (hpi). Pre-treatment with 3pRNA or IFN-α resulted in a significant reduction in the percent of IAV-infected A549 (Figure 1(Bi)) and LA-4 (Figure 1(Ci)) cells, as determined by assessing expression of newly synthesised viral NP within infected cells. Next, A549 and LA-4 cells treated with ctrl RNA or 3pRNA were subsequently infected with a high MOI (10 PFU/cell) of IAV HKx31 to assess single cycle virus growth at 2 versus 24 hpi (Figure 1(Bii,Cii)) or with a low MOI (0.1 PFU/cell) in the presence of exogenous trypsin to assess multicycle virus growth over time (Figure 1(Biii,Ciii)). These studies confirmed that pre-treatment with 3pRNA 24 h prior to virus infection resulted in potent inhibition of subsequent virus growth in both human and mouse airway cell lines. Together, these data confirm that pre-treatment with 3pRNA results in potent ISG induction and that this correlates with inhibition of IAV infection and growth in human and mouse airway epithelial cells. Of note, LA-4 cells express the *Mx1* gene but do not express a functional Mx protein.

### 3.2. RIG-I Agonist Treatment Induces ISG Expression in Primary Lung Cells from Mice Which Do and Do Not Express a Functional Mx1 Protein, Where Functional Mx Expression Results in Inhibition of IAV Infection In Vitro

**Figure 2:**
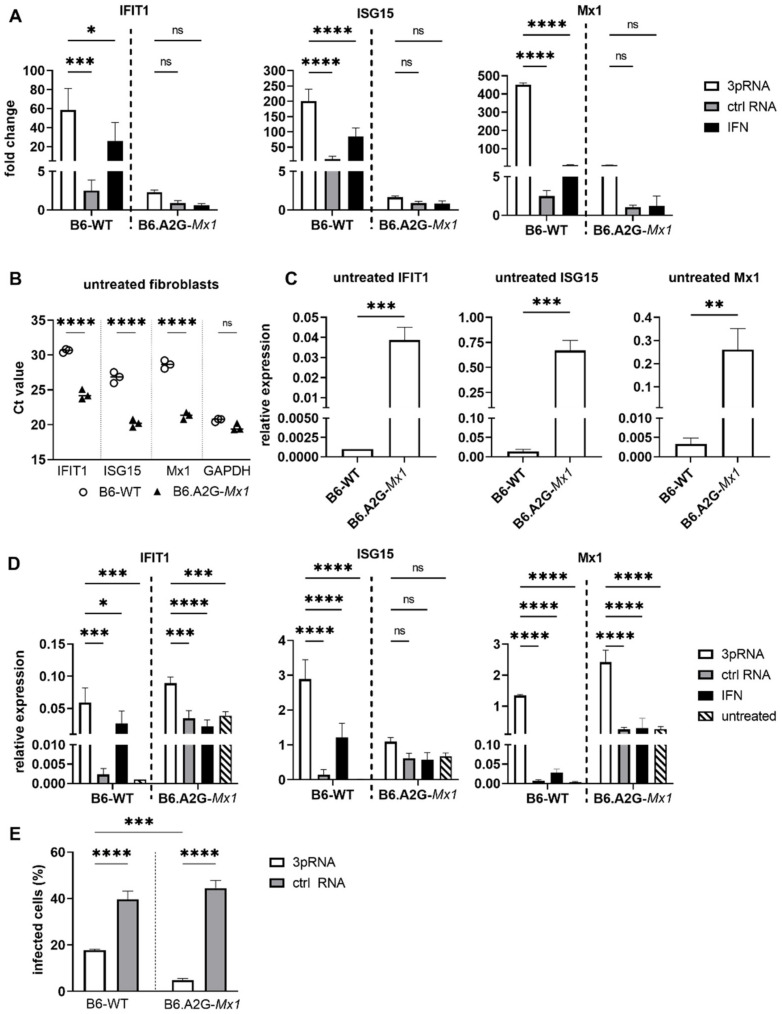
RIG-I agonist-mediated ISG induction in B6.A2G-*Mx1* and B6-WT primary fibroblasts. Cultures of primary mouse lung fibroblast (MLF) were transfected with RIG-I agonist (3pRNA, 200 ng/mL) or control RNA (ctrl RNA, 200 ng/mL), or treated with IFN-α (10^4^ units/mL). After 24 h, total RNA was isolated and the expression of ISGs was assessed by qRT-PCR. (**A**) Expression was normalized to GAPDH and is shown as fold induction relative to untreated cells. (**B**) Ct values were determined in untreated cells. (**C**) ISG expression relative to GAPDH was determined in untreated cells by calculating 2-ΔCt values. (**D**) ISG expression relative to GAPDH was determined in 3pRNA- (white), ctrl RNA- (grey) or IFN-α-treated cells (black) or in untreated samples (black and white stripes). (**E**) Cultures of primary mouse lung fibroblast (MLF) from B6-WT and B6.A2G-*Mx1* mice were transfected with RIG-I agonist (3pRNA, 200 ng/mL) or control RNA (ctrl RNA, 200 ng/mL) and, 24 h later, cells were inoculated for 1 h at 37 °C with IAV (strain HKx31, H3N2) at MOI of 10. Cells were incubated an additional 7 h, then fixed and stained for intracellular expression of the viral NP and analysed by flow cytometry. Data in (**A**,**C**–**E**) represent the mean (±SD), data in (**B**) represent the median and are representative of 2 experiments in triplicate samples, using fibroblasts sourced from different animals in each experiment. A two-way ANOVA with Bonferroni’s multiple comparison test was performed in (**A**,**B**,**D**) to compare 3pRNA to other conditions. An unpaired Student’s *t*-test was performed in (**C**) and multiple Student’s *t*-tests in (**E**) to compare B6-WT to B6.A2G-*Mx1* cells. * = *p* < 0.05; ** = *p* < 0.01; *** = *p* < 0.001; **** = *p* < 0.0001; ns = not significant.

Previous studies by Coch et al. reported that i.v. 3pRNA treatment of mice expressing a functional Mx1 protein induced potent protection against IAV [11]. As Mx1 is an ISG and a potent inhibitor of IAV, we first aimed to compare 3pRNA-mediated ISG induction and protection against IAV using primary mouse lung fibroblasts (MLF) generated from C57BL/6 mice which do (B6.A2G-*Mx1*) and do not (B6-WT) express a functional Mx1 protein [32]. First, MLF were assessed for ISG induction 24 h after treatment with 3pRNA, ctrl RNA or recombinant mouse IFN-α. Both 3pRNA and IFN-α induced upregulation of IFIT1, ISG15 and Mx1 in B6-WT MLF, whereas very little ISG induction was detected using B6.A2G-*Mx1* cells (Figure 2A). In this analysis (Figure 2A), ISG expression was determined, as for Figure 1A, using the 2^−ΔΔCt^ method which determines the fold change in ISG comparing test conditions relative to control (unstimulated) conditions, which is then normalised to the GAPDH housekeeping gene [30]. However, raw data indicated that Ct values for all ISGs tested were markedly lower in untreated samples from B6.A2G-*Mx1* compared to B6-WT cells whereas values for GAPDH were very similar (Figure 2B). Therefore, we focused on comparison of untreated samples from each mouse strain (i.e., the constitutive ISG expression) by calculating the relative expression, defined as the difference in Ct values of each ISG relative to GAPDH (2^−ΔCt^) [30]. Using this analysis, we confirmed that constitutive expression of each ISG was significantly higher in B6.A2G-*Mx1* cells compared to B6-WT cells (Figure 2C).

Next, we determined the relative IFIT1, ISG15 and Mx1 expression for B6-WT and B6.A2G-*Mx1* cells after 3pRNA, ctrl RNA or mouse IFN-α treatment. In cells from both B6-WT and B6.A2G-*Mx1* mice, the highest relative expression of each ISG was observed following 3pRNA treatment (Figure 2D). While relative expression after 3pRNA was similar for IFIT1 in B6-WT and B6.A2G-*Mx1* cells, ISG15 values were higher in B6-WT cells and Mx1 values were higher in B6.A2G-*Mx1* cells. Together, these data indicate that the increased constitutive expression of ISGs in unstimulated B6.A2G-*Mx1* cells likely contributes to the relatively modest increase observed after 3pRNA treatment when analysed using the 2^−ΔΔCt^ method.

Finally, MLF from B6-WT and B6.A2G-*Mx1* mice were treated with 3pRNA or ctrl RNA and 24 h later, were infected with HKx31 at MOI of 10. Pre-treatment of B6-WT MLF with 3pRNA resulted in a significant reduction in the percentage of IAV-infected cells at 8 hpi and this reduction was even more pronounced following 3pRNA pre-treatment of B6.A2G-*Mx1* cells (Figure 2E).

### 3.3. A Single Intravenous Injection with 3pRNA Provides Potent Protection against Subsequent IAV Infection in Both B6-WT and B6.A2G-Mx1 Mice While Intranasal Prophylaxis Provides Limited Protection Only in B6.A2G-Mx1 Mice

Studies to date investigating 3pRNA-mediated protection of mice against IAV infection have utilised the intravenous (i.v.) route of administration [8,9,10,11]. Moreover, i.v. and intraperitoneal injections were used to demonstrate 3pRNA-mediated protection against SARS-CoV-2 [12,13] and CHIKV [7], respectively. Given that IAV is a respiratory virus, our initial studies in mice compared the effectiveness of a single intranasal (i.n). versus i.v. treatment in providing protection against subsequent IAV infection. In preliminary studies, we confirmed that a single i.v. injection (12.5 μg in 200 μL) or i.n. inoculation (12.5 μg in 50 μL) of 3pRNA or ctrl RNA alone did not induce significant weight loss in B6-WT mice (data not shown).

**Figure 3:**
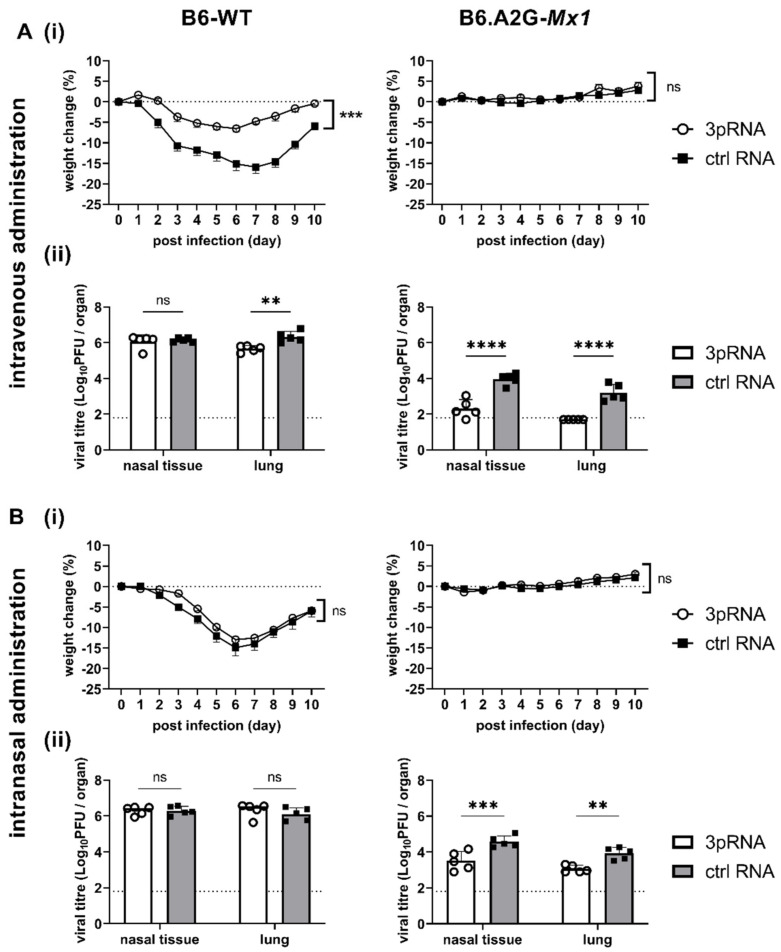
Intravenously applied RIG-I agonists provide superior protection from IAV HKx31 infection than intranasal treatment in B6.A2G-*Mx1* mice. (**A**) B6-WT (left) and B6.A2G-*Mx1* mice (right) received a single i.v. (**A**) or i.n. (**B**) treatment of 12.5 μg RIG-I agonist (3pRNA) or control RNA (ctrl RNA) and, 24 h later, were infected by the i.n. route with 10^4^ PFU of HKx31 (H3N2) in 50 μL of PBS. (**A**)/(**B**)(i) Mice were monitored daily and body weight was recorded. Data show the mean percent (±SEM) of weight change over time relative to original body weight (*n* = 5/group). (**A**(ii),**B**(ii)) At 5 dpi, nasal tissue and lungs were harvested, homogenized and virus titres in clarified homogenates were determined by plaque assay on MDCK cells (*n* = 5/group). Data are expressed as mean ± SD. Dashed lines represent the limit of detection of the plaque assay. Samples below the detection limit (<1.8 Log_10_PFU/organ) were assigned a value of 1.7 Log_10_PFU/organ for statistical analysis. In (**A**)/(**B**)(i) Student’s *t*-test was performed to compare weight loss after 3pRNA or ctrl RNA treatment. In (**A**(ii),**B**(ii)) a Two-way ANOVA with Bonferroni’s multiple comparison test was performed to compare viral titres after 3pRNA or ctrl RNA treatment. ** = *p* < 0.01; *** = *p* < 0.001; **** = *p* < 0.0001; ns = not significant.

Therefore, B6-WT and B6.A2G-*Mx1* mice received a single i.v. or i.n. treatment of 3pRNA or ctrl RNA (as described above) and, 24 h later, were infected by the i.n. route with 10^4^ PFU of the mouse-adapted IAV strain HKx31. First, weight loss was assessed over 10 days. After i.v. prophylaxis (Figure 3(Ai), left panel), B6-WT mice that received ctrl RNA showed marked weight loss over time whereas 3pRNA injection resulted in only modest weight loss. In contrast, IAV infection of B6.A2G-*Mx1* mice did not result in significant weight loss, irrespective of prophylaxis with 3pRNA or ctrl RNA (Figure 3(Ai), right panel), consistent with previous studies reporting that expression of a functional Mx1 protein was associated with resistance to IAV infection [15,16,18]. In subsequent experiments, mice were pre-treated as described above and then killed at day 5 post-infection (dpi) to determine viral loads in nasal tissues and in the lungs. After i.v. prophylaxis, 3pRNA was associated with a significant reduction in virus titres in the lungs, but not in the nasal tissues, of B6-WT mice (Figure 3(Aii), left panel). Whilst no differences were observed in weight loss, 3pRNA prophylaxis of B6.A2G-*Mx1* mice resulted in significantly reduced virus titres in both lungs and nasal tissues (Figure 3(Aii), right panel). Thus, consistent with previous reports, a single i.v. injection of 3pRNA to mice which do [11] or do not [8,9,10] express a functional Mx1 protein resulted in protection from subsequent challenge with IAV.

Following prophylaxis via the i.n. route, B6-WT mice showed marked weight loss and no significant differences were observed between animals pre-treated with ctrl RNA or 3pRNA (Figure 3(Bi), left panel). Again, IAV infection of B6.A2G-*Mx1* did not result in any significant weight loss over time. Thus, when examining weight loss after IAV infection we observed that a single i.v., but not an i.n., treatment with 3pRNA provided some protection against subsequent IAV challenge in B6-WT mice. In contrast, IAV infection of B6.A2G-*Mx1* did not result in significant loss of body weight, irrespective of prophylaxis by i.v. or i.n. routes (Figure 3(Ai,Bi), right panels). After i.n. treatment, no differences were noted in virus titres recovered from the nasal tissues or the lungs of B6-WT mice which received 3pRNA or ctrl RNA (Figure 3(Bii), left panel). For B6.A2G-*Mx1* mice, significantly reduced levels of virus were recovered both from nasal tissue and lung (Figure 3(Bii), right panel), however viral titres after 3pRNA prophylaxis via the i.n. route were markedly higher compared to the i.v. route, indicating a superior protective effect against IAV infection when using the i.v. route of administration.

### 3.4. Intravenous Injection of RIG-I Agonist Induces ISG Expression in the Lungs of B6-WT and B6.A2G-Mx1 Miceconfirm

Our data showed that i.v., but not i.n., prophylaxis of B6-WT mice with 3pRNA resulted in protection against subsequent challenge with IAV strain HKx31. Inhibition of IAV replication in the airways of B6.A2G-*Mx1* mice was more potently inhibited after i.v. delivery of 3pRNA prior to infection. Given that our in vitro studies demonstrated that treatment of mouse cells with RIG-I agonist induced upregulation of ISGs and protection against IAV infection and growth we next assessed ISG induction in the lungs following i.v. prophylaxis of mice with 3pRNA. Therefore, naïve B6-WT and B6.A2G-*Mx1* mice received a single i.v. injection with 3pRNA or ctrl RNA and, 1 or 5 days later, animals were killed and lungs removed for subsequent isolation of RNA. Untreated animals were also included for comparison in these experiments.

**Figure 4:**
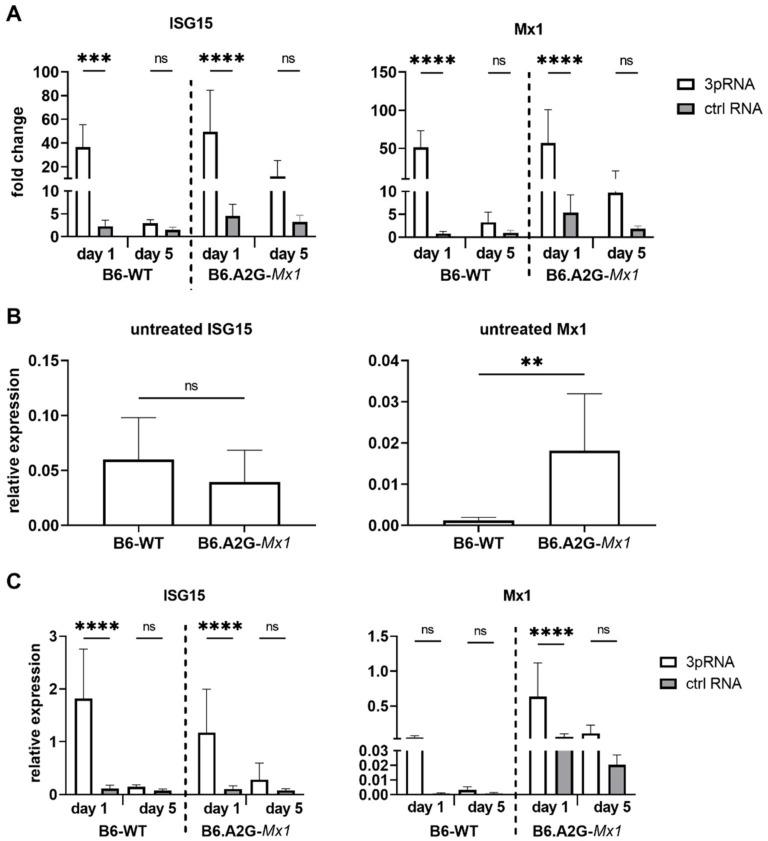
RIG-I agonist-mediated ISG induction in B6.A2G-*Mx1* and B6-WT mice. B6-WT and B6.A2G-*Mx1* mice received a single i.v. treatment of 12.5 μg RIG-I agonist (3pRNA) or control RNA (ctrl RNA). After 1 day or 5 days, total RNA was isolated and the expression of ISGs was assessed by qRT-PCR. (**A**) Expression was normalized to GAPDH and is shown as fold induction relative to untreated cells. (**B**) ISG expression relative to GAPDH was determined in untreated cells by calculating 2^−ΔCt^ values. (**C**) ISG expression relative to GAPDH was determined in 3pRNA- (white bars), ctrl RNA- (grey bars) 1 or 5 days after treatment. Data represent the mean (±SD) from triplicate samples and are pooled from 2 experiments. A two-way ANOVA with Bonferroni’s multiple comparison test was performed to compare 3pRNA to other conditions in (**A**,**C**). An unpaired Student’s *t*-test was performed in (**B**) to compare B6-WT to B6.A2G-*Mx1* mice. ** = *p* < 0.01; *** = *p* < 0.001; **** = *p* < 0.0001; ns = not significant.

As for primary MLF, qRT-PCR data for ISG15 and Mx1 expression was first analysed using the 2^−ΔΔCt^ method to determine the fold change in each ISG after test conditions relative to unstimulated conditions, which were then normalised to GAPDH. These analyses demonstrated that ISG15 and Mx1 were potently and significantly induced in the lungs of B6-WT and B6.A2G-*Mx1* mice 1 day after receiving 3pRNA- compared to ctrl RNA (Figure 4A). At 5 days after prophylaxis, ISG levels were no longer significantly increased at the mRNA level in mice that received 3pRNA from either mouse strain, although we did observe a trend for upregulated expression of ISG15 and Mx1 in lungs from B6.A2G-*Mx1* mice.

When examining Ct values for ISG expression in lungs from untreated mice, it was noted that Ct values for ISG15 were very similar between B6-WT and B6.A2G-*Mx1* mice whereas Ct values for Mx1 were markedly lower in lungs from B6.A2G-*Mx1* mice compared to B6-WT mice (data not shown). Analysis of the relative expression of ISG compared to GAPDH using the 2^−ΔCt^ method confirmed significantly increased expression of Mx1, but not ISG15, in B6.A2G-*Mx1* lungs (Figure 4B). We also determined the relative expression 1 and 5 days after i.v. injection with 3pRNA or ctrl RNA. In both B6-WT and B6.A2G-*Mx1* mice, the highest relative expression of each ISG was observed in mice receiving 3pRNA prophylaxis (Figure 4C). While relative expression after 3pRNA were similar for ISG15 in B6-WT and B6.A2G-*Mx1* mice, Mx1 values were higher in B6.A2G-*Mx1* mice. These findings are consistent with relative expression patterns in primary MLF isolated from B6-WT and B6.A2G-*Mx1* mice following 3pRNA treatment in vitro (Figure 2D). Together, these data indicate that a single i.v. injection with RIG-I agonist induces ISG mRNA expression in the lung tissue of both B6-WT and B6.A2G-*Mx1* mice but that this induction is short-lived and does not remain significantly upregulated 5 days after prophylaxis in either mouse strain. Of note, our in vivo studies confirm increased constitutive expression of Mx1 in lungs of B6.A2G-*Mx1* compared to B6-WT mice.

### 3.5. No Major Differences in Immune Cell Recruitment and Cytokine and Chemokine Release in the Lung after i.v. Prophylaxis of B6-WT and B6.A2G-Mx1 Mice with 3pRNA

In addition to ISG induction, we aimed to determine if i.v. 3pRNA prophylaxis modified the mouse airways in other ways prior to IAV infection. Therefore, B6-WT and B6.A2G-*Mx1* received i.v. injection with 3pRNA or ctrl RNA and, 24 h later, bronchoalveolar lavages (BALs) were performed and immune cell infiltrates characterised by flow cytometry. We did not record significant differences in the number of total immune (CD45+) cells, or in numbers of neutrophils (CD11b+, Ly6G+), eosinophils (CD11b+, Siglec-F+, CD11c−, CD64−), NK cells (CD3−, NK1.1+), alveolar macrophages (CD11b+, Siglec-F+, CD11c+, CD64+) and pan-macrophages (CD11b+, Siglec-F−, CD64+), or in CD4+CD3+ or CD8+CD3+ T lymphocytes in BAL from 3pRNA or ctrl RNA treated animals in either mouse strain (data not shown).

Next, we used a multiplex cytometric bead array (CBA) to determine levels of inflammatory cytokines and chemokines in cell-free BAL fluid of naive mice and of mice 24 h after injection with 3pRNA or ctrl RNA. Most inflammatory mediators tested were below detection limit and/or not significantly upregulated after 3pRNA, including IFN-γ, CXCL1, TNF-α, CCL2, IL-12 p70, CCL5, IL-1β, GM-CSF, IL-10, IFN-β, IFN-α and IL-6 (data not shown). However, 3pRNA injection was associated with a significant upregulation of CXCL10 relative to naive mice or ctrl RNA-treated mice (B6-WT: 3pRNA 1310.5 ± 922.4 SD ctrl RNA: 97.7 ± 11.9 SD, untreated: 13.1 ± 12.9 SD; B6.A2G-*Mx1*: 3pRNA 7272.7 ± 600.0 SD, ctrl RNA: 51.1 ± 52.6 SD, untreated 0.1 ± 0 SD). While there was a trend for enhanced CXCL10 induction by 3pRNA in B6-WT mice compared to B6.A2G- *Mx1* mice, this was not significant. Overall, these studies indicate that no major differences in inflammatory cells and/or mediators were detected in the BAL of B6-WT and B6.A2G-*Mx1* mice 1 day after i.v. injection of 3pRNA.

### 3.6. A functional Mx1 Is Required for Potent and Long-Lasting RIG-I Agonist-Mediated Protection against IAV in Primary Mouse Fibroblasts

**Figure 5:**
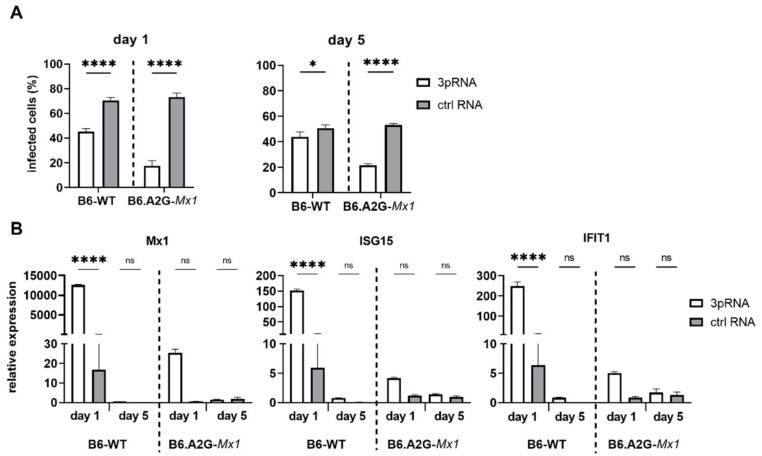
Treatment of primary mouse lung fibroblasts from B6-WT or B6.A2G-*Mx1* mice with RIG-I agonists inhibits IAV and induces Mx1-dependent long-term protection. Primary mouse lung fibroblasts (MLF) were transfected with RIG-I agonist (3pRNA, 200 ng/mL) or control RNA (ctrl RNA, 200 ng/mL). (**A**) At 1 or 5 days after treatment cells were infected with HKx31 (MOI = 50), then fixed and stained for intracellular expression of the IAV NP at 8 hpi. Cells were analysed by flow cytometry. (**B**) RNA was harvested either 1 or 5 days after 3pRNA or ctrl RNA treatment. Expression of Mx1, ISG15 and IFIT1 was assessed by qRT-PCR. Expression was normalized to GAPDH and expressed as fold induction relative to untreated cells. Data represent the mean (±SD) from triplicate samples and are representative of 2 independent experiments. In (**A**,**B**), a two-way ANOVA with Bonferroni’s multiple comparison test was performed to compare 3pRNA and ctrl RNA treatment. * = *p* < 0.05; **** = *p* < 0.0001; ns = not significant.

Given that prophylaxis of B6.A2G-*Mx*1 mice with 3pRNA was associated with potent and long-lasting protection against IAV challenge [11], MLF isolated from B6-WT or B6.A2G-*Mx1* mice were transfected with 3pRNA or ctrl RNA and then infected with IAV either 1 or 5 days later. The percentage of IAV-infected cells was then determined at 8 hpi to assess susceptibility to virus infection. As seen in Figure 5A, 3pRNA treatment 1 day prior to infection significantly reduced the percentage of IAV-infected cells in both B6-WT and B6.A2G-*Mx1* MLF. Notably, 3pRNA-mediated inhibition was more potent in B6.A2G-*Mx1* cells. Even 5 days after 3pRNA treatment, IAV infection was potently inhibited in B6.A2G-*Mx1* cells whereas the inhibitory effect was significant, although quite modest, in B6-WT cells. Thus, 3pRNA pre-treatment of MLF in vitro results in a more potent and long-lasting inhibitory effect when using cells from mice expressing a functional Mx1 protein.

To determine if long-term protection from IAV infection correlated with enhanced ISG expression, levels of ISG15, Mx1 and IFIT1 mRNA in B6-WT and B6.A2G-*Mx1* MLF were determined either 1 or 5 days after 3pRNA or ctrl RNA treatment (Figure 5B). Using the 2-ΔΔCt method, 3pRNA induced ISG expression in B6-WT and, to lesser extent, in B6.A2G-*Mx1* MLF at day 1 post-treatment, similar to results in Figure 2A. Again, B6.A2G-*Mx1* MLF showed higher constitutive ISG expression in unstimulated conditions compared to B6-WT cells when 2-ΔCt ratios were examined (data not shown). Of interest, 5 days after 3pRNA-treatment levels of ISG15, Mx1 or IFIT1 were not significantly higher than ctrl RNA-treated cells for either B6-WT or B6.A2G-*Mx1* MLF. Together, these data highlight the importance of a functional Mx1 protein for RIG-I agonists to mediate potent and long-lasting protection against IAV infection in primary MLF in vitro. However, while B6.A2G-*Mx1* fibroblasts showed higher constitutive expression of ISG mRNA, we did not find a direct correlation between long-lasting protection against IAV infection in B6.A2G-*Mx1* MLF and maintenance of 3pRNA-induced upregulation of the ISGs examined.

### 3.7. A Single Prophylactic Injection with 3pRNA Results in Potent and Long-Lasting Protection in B6.A2G-Mx1 However, Not B6-WT Mice following Infection with the Virulent Mouse-Adapted PR8 IAV Strain

**Figure 6:**
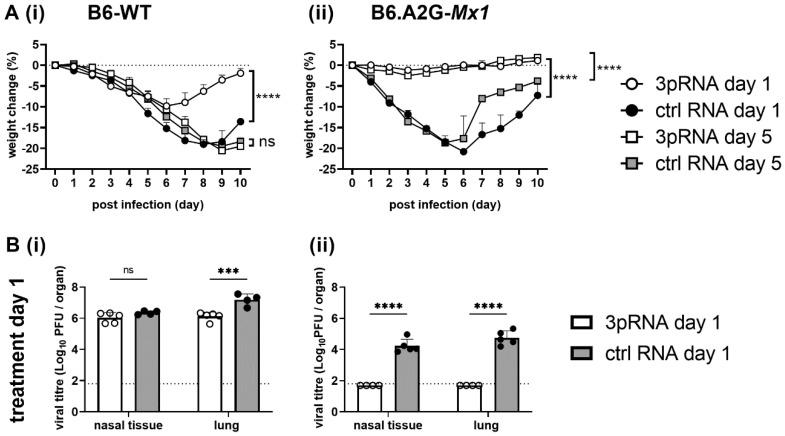

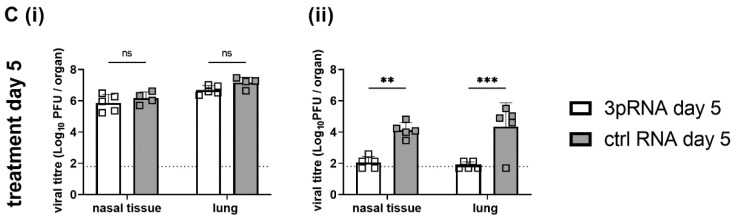
After a single intravenous injection of mice with RIG-I agonist, expression of a functional Mx1 protein is critical for potent and long-lasting protection from subsequent IAV challenge. B6-WT and B6.A2G-*Mx1* mice received a single i.v. injection of 12.5 μg RIG I agonist (3pRNA) or control RNA (ctrl RNA). At either 1 or 5 days after treatment mice were infected by the i.n. route with 10^2^ PFU (B6-WT) or 10^6^ PFU (B6.A2G-*Mx1*) of IAV strain PR8 (H1N1) in 50 μL of PBS. (**A**) Mice were monitored daily, and data shows the mean percent (± SEM) of weight change over time determined relative to original body weight. (**B**,**C**) Nasal tissues and lungs were harvested 5 days after infection, homogenized and virus titres in clarified homogenates were determined by plaque assay on MDCK cells. Data are expressed as mean ± SD (*n* = 4–5/group). Dashed lines represent the limit of detection of the plaque assay. Samples below the detection limit (<1.8 log_10_PFU/mL) were assigned a value of 1.7 log_10_PFU/mL for statistical analysis. (**A**) Student’s *t*-test was performed to compare weight loss after 3pRNA or ctrl RNA treatment. (**B**,**C**) Two-way ANOVA with Bonferroni’s multiple comparison test was performed to compare weight loss and viral titres after 3pRNA or ctrl RNA treatment. ** = *p* < 0.01; *** = *p* < 0.001; **** = *p* < 0.0001; ns = not significant.

Next, we infected B6-WT and B6.A2G-*Mx1* mice with the mouse virulent PR8 IAV (H1N1) strain. A higher inoculum dose was used to infect B6.A2G-*Mx1* compared to B6-WT animals, to achieve comparable weight loss between the two mouse strains. In these experiments, mice were challenged with 10^2^ PFU (B6-WT) or 10^6^ PFU (B6.A2G-*Mx1*) of PR8 either 1 or 5 days after injection with ctrl RNA or 3pRNA to assess the longevity of any protective responses observed. For B6-WT mice, 3pRNA prophylaxis of B6-WT mice 1, but not 5, days prior to PR8 challenge resulted in less pronounced weight loss compared to animals receiving ctrl RNA (Figure 6(Ai)). This correlated with a reduction in virus titres in the lungs of mice treated with 3pRNA at 1 (Figure 6(Bi)), but not 5 days (Figure 6(Ci)), prior to virus challenge and no significant differences were noted in titres in nasal tissue following either regimen (Figure 6(Bi,Ci)). For B6.A2G-*Mx1* mice, animals injected with ctrl RNA lost 15–20% of their original body weight over time, whereas 3pRNA injection either 1 or 5 days prior to infection resulted in negligible weight loss (Figure 6(Aii)). Moreover, 3pRNA prophylaxis 1 or 5 days prior to infection also resulted in significant reductions in virus titres in nasal tissues and lungs (Figure 6(Bii,Cii)). Together, these data indicate that RIG-I agonists mediate inhibition of IAV in mice and that a functional Mx1 protein is required for induction of potent and long-lasting protection against IAV both in vitro and in vivo.

## 4. Discussion

To date, most in vivo studies evaluating the antiviral function of RIG-I agonists against IAV have been performed using laboratory mouse strains such as C57BL/6 and BALB/c which do not express a functional Mx1, an ISG protein known to mediate potent anti-IAV activity [33]. These studies report that prophylactic i.v. administration of RIG-I agonists 24 h prior to infection combined with a second dose on the day of infection [8,9], or alternatively, combined with three subsequent doses post infection [10], can provide some protection from subsequent challenge with different IAV, including H5N1 strains. In general terms, these RIG-I agonist treatment regimens improved overall survival rates, although only modest differences in IAV-induced weight loss were noted when compared to control animals [8,9]. Moreover, injection with RIG-I agonist also resulted in a relatively modest, but significant, reduction in virus replication in the lungs that could be enhanced by multiple doses [8,9,10]. Subsequent studies by Coch et al. used mice that expressed a functional Mx1 to demonstrate potent and long-lasting protection from IAV infection following a single injection of 3pRNA [11].

Herein, we have performed a direct comparison of the effects of 3pRNA on subsequent IAV infection using congenic mice that do (B6.A2G-*Mx1*) or do not (B6-WT) express a functional Mx1. The mouse lines used were derived from breeding of heterozygous littermates to ensure that the impact of Mx1 expression alone could be examined. While i.v. injection of 3pRNA prior to IAV infection was protective in both mouse strains (as assessed by IAV-induced weight loss and virus replication in the airways at day 5 post-infection), it was particularly potent and long-lasting in mice expressing a functional Mx1 protein. This is likely due to the direct antiviral effects of Mx1 against IAV, where it is known to be a potent inhibitor of viral transcription [34,35,36]. Consistent with this, data obtained using ctrl RNA-treated animals confirmed B6.A2G-*Mx1* mice to be intrinsically more resistant to IAV infection, as evidenced by negligible weight loss and reduced viral titres 5 dpi following infection with an equivalent virus dose. These findings are consistent with numerous studies that have reported the resistance of mice expressing functional Mx1 to IAV infection, including by H5N1 and pandemic IAV strains [15,18]. For these reasons, B6.A2G-*Mx1* mice were challenged with a much higher (10,000×) dose of the virulent mouse-adapted PR8 strain to demonstrate that 3pRNA mediated potent and long-lasting inhibitory activity against IAV.

To date, studies reporting the antiviral activity of 3pRNA treatment against IAV and other viruses in vitro have used human cells, including the A549 airway epithelial cell line [7,8,9,10], as well as human monocyte-derived DCs [9] or human myeloid cells [7]. Herein, we also demonstrate effective ISG induction and subsequent inhibition of IAV replication in vitro using mouse LA-4 airway epithelial cells, as well as primary mouse lung fibroblasts isolated from B6-WT and B6.A2G.Mx1 mice. Moreover, we have correlated these in vitro findings with in vivo data using the mouse model of IAV infection. While pre-treatment with 3pRNA induced ISG upregulation in all mouse cells tested (LA-4 and MLF from B6-WT and B6.A2G-*Mx1* mice), inhibition of IAV infection was particularly potent in MLF isolated from B6.A2G-*Mx1* mice. Moreover, subsequent analysis using the 2^−ΔCt^ method (i.e., to examine ISG expression relative to GAPDH housekeeping gene) indicated that constitutive ISG expression was markedly elevated in B6.A2G-*Mx1* cells and this likely contributes to the modest further upregulation in expression of ISGs following 3pRNA treatment when analysed using the 2^−ΔΔCt^ method. In addition to increased constitutive expression of Mx1 mRNA, it is interesting to note that constitutive levels of IFIT1 and ISG15 were also higher in MFL from B6.A2G-*Mx1* mice although only Mx1, and not ISG15, were higher when analysing mRNA from mouse lung. Nonsense-mediated decay (NMD) is a quality control mechanism in eukaryotic cells whereby mRNAs with premature termination codons are degraded [37]. Given that aberrant mRNA transcripts with premature termination codons may be produced in B6-WT mice [38], this might contribute to the reduced mRNA transcript levels detected in the lungs and MLF of these mice when compared to B6.A2G-*Mx1* counterparts. However, our results also suggest that expression of a functional Mx1 might also modulate expression of other ISGs, particularly in MLF, although further studies are required to determine if constitutive expression of a broader panel of ISGs is enhanced in lungs and other tissues of B6.A2G-*Mx1* mice.

In addition to IAV, the antiviral function of murine Mx1 has been investigated in the context of a number of other viral infections. For example, Haller et al. found that congenic Balb/c and C57BL/6 mice expressing a functional (A2G) Mx1 were protected from lethal infection with two tick-borne members of the Orthomyxoviridae family, namely Thogoto Virus (THOV) and Dhori virus (DHOV) [39]. However, comprehensive studies comparing Mx1-expressing A2G mice with Mx1-deficient control mice demonstrated that expression of a functional Mx1 did not result in enhanced resistance to a range of other viruses including yellow fever virus, West Nile virus and mouse encephalomyocarditis virus [40], or to Newcastle disease virus, rabies virus or vesicular stomatitis virus [41]. Thus, while our in vitro (MLF cells) and in vivo (lungs from naïve mice) data suggest a tendency for enhanced constitutive expression of Mx1 and possibly some other ISGs in congenic B6.A2G-*Mx*1 mice, it is notable that expression of A2G Mx1 is associated with specific resistance to orthomyxoviruses and not with a generalised antiviral activity against diverse viruses.

Of interest, 3pRNA prophylaxis provided long-term protection (i.e., 5 days after treatment) against subsequent IAV infection in vitro (using MLF) or in vivo (i.n. infection) only in B6.A2G-*Mx1* mice, although when examining mRNA levels in MFL or mouse lung 5 days after treatment we did not detect evidence of upregulated Mx1 expression. These findings are curious and suggest that Mx1 protein expression might be rapidly upregulated and relatively stable in MLF and lung cells in B6.A2G-*Mx1* mice, such that functional Mx1 is able to mediate potent anti-IAV activity for many days after induction by 3pRNA. While attempts to quantitate endogenous Mx1 protein expression in MLF lysates by Western blot were not successful, we did generate LA-4 cells with doxycycline (dox)-induced expression of FLAG-tagged functional Mx1 and confirmed that protein levels were not markedly reduced 48 h after removal of dox [42]. In contrast, dox-inducible expression of other ISGs, namely IFITM1, IFITM2 and IFITM3, in A549 cells had returned to background levels 48 h after removal of dox [43]. While the mechanisms underlying induction of FLAG-tagged Mx1 by dox are not physiologically relevant and protein turnover is likely to vary depending on the particular protein and/or the cell type examined, it is tempting to speculate that a relatively slow turnover of Mx1 protein in MLF and in the lungs of B6.A2G-*Mx*1 might be an important factor contributing to the long-lasting and potent 3pRNA-mediated protection against IAV observed only in these mice.

It was interesting to note that i.v., rather than i.n. delivery of 3pRNA, was associated with more effective inhibition of IAV in both B6-WT and B6.A2G-*Mx1* mice. Although i.v. and i.n. delivery of 3pRNA to B6.A2G-*Mx1* mice resulted in significant reductions in viral loads in both the lungs and nasal tissues, only i.v. injection resulted in a modest, but significant, reduction in lung virus titres in B6-WT mice. Any impact of 3pRNA prophylaxis on virus replication in the nasal tissues was only observed in mice expressing a functional Mx1. These data indicate that a substantial proportion of PEI-formulated 3pRNA is delivered to parenchymal cells of the lung following i.v. (systemic) injection, although delivery to the nasal tissues appears less effective. In contrast, i.n. (local) delivery of 3pRNA to the airways resulted in modest (B6.A2G-*Mx1*) or negligible (B6-WT) effects on subsequent IAV replication in the respiratory tract. As RIG-I is expressed in almost all nucleated cells [44], this might represent an issue in effective delivery to the epithelium due to an inability to penetrate the mucus layer and/or degradation by enzymes present in airway fluids. However, it should be noted that PEI-formulated 3pRNA retained activity in the blood following i.v. injection and that PEI-formulated siRNA [45] and DNA [46] have been effectively delivered to mice via the i.n. route in other studies. After i.n. delivery, 3pRNA was also associated with modest, but significant, reductions in virus titres in the lungs and nasal tissues of B6.A2G-*Mx1* mice, providing evidence that at least some RIG-I agonist was delivered to airway cells via this route.

While we noted major differences in 3pRNA-mediated protection against IAV infection in B6-WT versus B6.A2G-*Mx1* mice, we did not detect major differences in the lung environment (i.e., cellular inflammation in BAL, inflammatory mediators in cell-free BAL) 24 h after naive mice received a single i.v. dose of 3pRNA. Overall, studies presented herein demonstrate that expression of a functional Mx1 is associated with enhanced RIG-I agonist-mediated inhibition of IAV infection in vitro and is the key determinant of the enhanced potency and duration of 3pRNA-mediated protection against IAV infection observed in B6.A2G-*Mx1* mice.

## Data Availability

Not applicable.
